# Lost paradise—anthropogenic pressure alters microbial functional diversity in an endangered endemic toad-habitat system

**DOI:** 10.1093/ismeco/ycaf239

**Published:** 2025-12-16

**Authors:** Philippe J R Kok, Magdalena Urbaniak

**Affiliations:** Department of Ecology and Vertebrate Zoology, Faculty of Biology and Environmental Protection, University of Lodz, Banacha 12/16, Łódź 97-237, Poland; Life Sciences, The Natural History Museum, Cromwell Road, London SW7 5BD, United Kingdom; Faculty of Biology and Environmental Protection, UNESCO Chair on Ecohydrology and Applied Ecology, University of Lodz, Banacha 12/16, Łódź 97-237, Poland

**Keywords:** amphibia, Biolog® EcoPlates™, human disturbance, microbiomes, *Oreophrynella*, Pantepui, tepui, tourism

## Abstract

Tourism-driven human activity is increasingly disrupting fragile and once-pristine ecosystems worldwide, as evidenced by coral reef degradation in the Great Barrier Reef, vegetation loss in the Himalayas, and, as demonstrated in this study, microbial shifts in isolated highland habitats such as tepui summits. Integrating field-based ecological, microbiological, and conservation perspectives, this study provides novel insights into how anthropogenic disturbance—particularly tourism—affects microbial functional diversity across interconnected environmental (soil) and host-associated (amphibian skin and faeces) compartments in a globally unique and poorly studied highland ecosystem, the summit of Roraima-tepui in Venezuela. Our results provide clear evidence that anthropogenic disturbance on the summit of Roraima-tepui reduces microbial functional diversity—by 59% in soil and by 21% and 14% in the skin and faecal microbiomes of the (near)endemic toad *Oreophrynella quelchii*, respectively—compared to pristine sites. Our findings raise significant concern, as alterations in microbial composition and functions could disrupt host immunity and disease resistance in this unique, insular, and ecologically fragile ecosystem, particularly given the recent detection of anthropogenic pathogen incursion in amphibian communities. Our results stress the need to better understand the link between the observed shift in the skin microbiome’s functional profiles in *O. quelchii* at summit sites most impacted by tourism and the recent emergence of the fungal pathogen *Batrachochytrium dendrobatidis* in the same environmental context. Our findings underscore the urgent need to mitigate human-induced pressures threatening the ecological integrity of the summit of Roraima-tepui, one of the world’s most fragile and irreplaceable montane habitats.

## Graphical Abstract

**Graphical Abstract:**
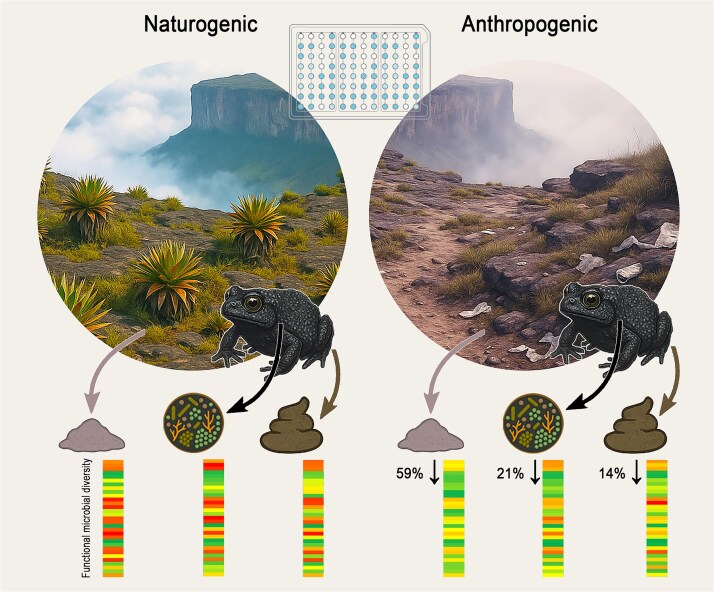

## Introduction

Human-driven environmental changes are causing unprecedented extinction rates among plants and animals, with far-reaching consequences for global biodiversity [1–3]. While the decline of visible species, biological communities, and habitats is well documented [1, 4], the effects of anthropogenic pressures on microbial life and host-microbiome interactions remain comparatively underexplored. Microorganisms, though invisible to the naked eye, are extraordinarily abundant with an estimated 10^30^ bacterial and archaeal cells across all environments on Earth [5], and are functionally diverse, playing foundational roles in biogeochemical cycles and ecosystem functioning [6]. These microbial systems underpin the essential life-support mechanisms of the biosphere. Importantly, anthropogenic disruption of microbiome-driven processes can reduce microbial biodiversity and metabolic activity, thereby diminishing ecosystem resilience and compromising the health of macroorganisms, such as amphibians [7–10], particularly under environmental stressors like climate change [11].

**Figure 1:**
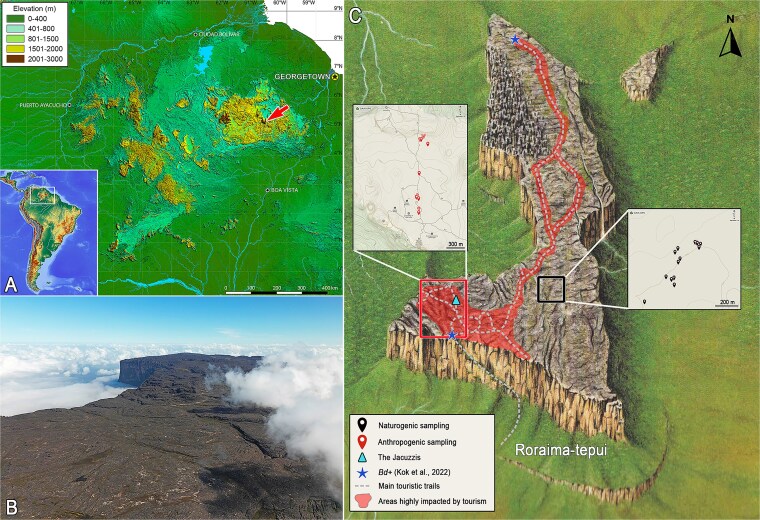
Overview of the study site and sampling localities. The Pantepui region and its location in northern South America (lower left inset), red arrow indicates the location of Roraima-tepui (A), drone image of the summit, looking north (B), sampling sites (C, image modified from [25]). Photo by P.J.R.K.

Global amphibian declines are driven by multiple, often interacting factors, most of which are anthropogenic [1]. Human-driven impacts now reach even remote environments like the summit of Roraima-tepui, an iconic continental-island ecosystem, where unregulated tourism is introducing rapid environmental change. Tepui summits are renowned for high endemism and ecological singularity, shaped by long-term geographic isolation, oligotrophic soils, and harsh climate. Reaching elevations of ca. 3000 m, they form part of the Pantepui region in the Guiana Shield’s highlands in northeastern South America [12], often dubbed as “*The Lost World*” (Fig. 1A and B). Tepui ecosystems have been model systems for studies of vertebrate speciation [13–16], climatic refugia [17, 18], biogeographic patterns [19, 20], and more recently, pathogen incursions in naïve amphibian communities [21]. Increasing tourism pressure [21–23] has been associated with the introduction of exotic plant species [22, 23], pathogenic bacteria such as *Helicobacter pylori* [24], and amphibian skin-associated pathogens [21] on the summit of Roraima-tepui, posing a growing threat to the health and integrity of the tepuis’ endemic flora and fauna.

Of particular concern is the endemic toad *Oreophrynella quelchii*, which is found only on two tepuis (Roraima and Wei-Assipu) at 2200–2810 m in Venezuela, Guyana, and Brazil [26]. Despite high local abundance on Roraima (estimated at ca. 12 million individuals [27]), *O. quelchii* is listed as Endangered by the IUCN due to its narrow distribution and increasing anthropogenic disturbance, including projected climate change [28].

Although sanitation facilities are mandated for tourists on the summit of Roraima-tepui, enforcement for porters and guides, who often outnumber tourists, is missing and improper waste disposal is frequent due to limited infrastructure. Given the small size of the summit (ca. 35 km^2^) and the oligotrophic nature of its soils, human waste poses a significant threat to this fragile ecosystem by disrupting nutrient balance and potentially impacting local biodiversity. Since soil bacteria play essential roles in processes such as organic matter decomposition, nutrient cycling, and pathogen suppression [29], disturbances to soil microbial communities may propagate through interconnected ecological networks, potentially altering host-associated microbiomes. Amphibian skin, characterised by exceptional permeability and continuous moisture, constitutes a primary interface with the environment and a major route for pathogen transmission. The associated microbiome forms a critical component of host defense, maintaining microbial homeostasis and contributing to disease resistance [30]. These functions are especially important given the global threat posed by the fungal pathogens *Batrachochytrium dendrobatidis* (Bd) and *B. salamandrivorans* (Bsal), which are responsible for widespread amphibian declines [31, 32]. Human disturbance has been shown to reduce amphibian skin microbial diversity and alter community structure, potentially weakening resistance to infection [7, 8]. While microbial taxonomic diversity has received most research attention, functional diversity through community-level physiological profiling (CLPP) provides deeper insight into the ecological roles of host-associated microbiomes and their capacity for disease resistance and environmental adaptation [33]. Functional diversity profiling using the Biolog® EcoPlate™ method enables the detection of changes in the metabolic potential of microbial communities [34]. Each plate contains three replicated sets of 31 preselected carbon substrates representative of major biochemical groups (amines - A, amino acids- AA, carboxylic acids - CA, complex carbon sources - CCS, carbohydrates - CH, phosphate carbon - PC) and employs a tetrazolium redox dye as an indicator of microbial respiration. When microorganisms metabolise these substrates, the reduction of the dye produces a measurable colour change, generating a characteristic metabolic fingerprint that reflects the functional capabilities of the microbial community. Several key indices are derived from these data, including the average well colour development (AWCD), Shannon diversity index (H′), Shannon evenness index (E), and substrate richness (S) [35–37]. Compared to molecular and culture-based methods, which are often labour-intensive and require specialised infrastructure, the Biolog® EcoPlate™ technique provides a rapid, cost-effective, and standardised approach for assessing microbial functional diversity [38]. This method has been extensively used to evaluate the impacts of anthropogenic disturbances on microbial communities in soils and sediments (e.g. [39–43]), as well as in plant-associated (e.g. [44, 45]) and animal-associated microbiomes, including gut and faecal microbial consortia (e.g. [46–50]). It has also been applied to characterise microbial metabolic dynamics in manure composting and wastewater systems (e.g. [51, 52]). Importantly, functional diversity profiles generated by the Biolog® EcoPlate™ method have shown strong agreement with results obtained from molecular and biochemical analyses such as high-throughput sequencing, denaturing gradient gel electrophoresis (DGGE), and phospholipid fatty acid (PLFA) profiling [43, 53], while offering a cost-effective and accessible alternative, particularly suited for remote or resource-limited settings [34].

Motivated by field observations of habitat degradation and pollution, particularly the accumulation of human waste, on the summit of Roraima, we investigated the functional diversity of environmental and host-associated microbial communities as indicators of anthropogenic impact. We hypothesised that sites exposed to human disturbance would exhibit altered or reduced microbial functional diversity in both summit soils and the skin and faecal microbiomes of *O. quelchii*, potentially destabilising key ecosystem processes and impairing host immune function. To test this hypothesis, we employed the Biolog® EcoPlates™ method [54] to assess the functional diversity of microbial communities in Roraima-tepui soils and in the skin and faecal microbiota of *O. quelchii,* comparing naturogenic (undisturbed) and anthropogenically impacted sites. To our knowledge, this is the first application of Biolog® EcoPlates™ to amphibian-associated microbiomes.

By focusing on a remote, highly isolated montane ecosystem, this study contributes to a broader understanding of microbiome disruption and host vulnerability under anthropogenic pressure, with potential implications for biodiversity conservation in similarly fragile environments.

## Materials and methods

### Study sites

**Figure 2:**
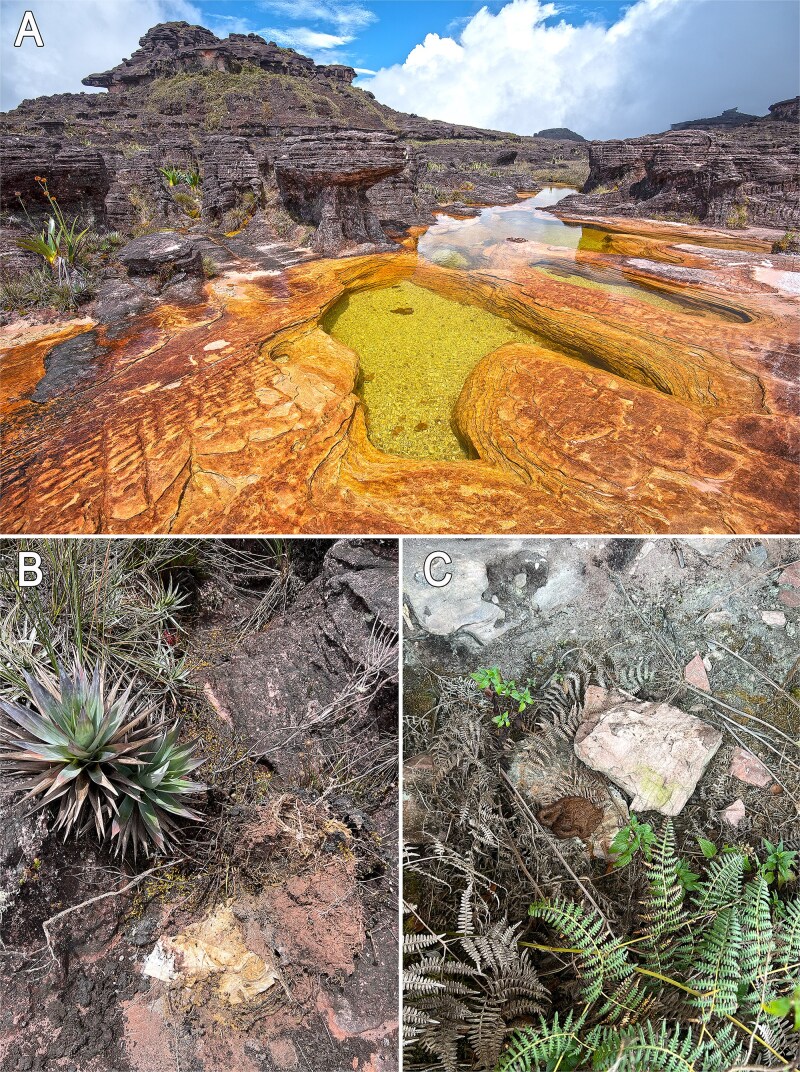
Human-impacted area on the tepui summit. “The Jacuzzis” a famous natural feature on the summit widely visited by tourists, who often bathe or wade in its waters, unaware that they literally swim in a toilet bowl (A), human waste around The Jacuzzis (B–C). Photos by P.J.R.K.

Our sampling was conducted in February–March 2025, on the summit of Roraima-tepui, one of the highest tepuis with a maximum elevation of 2810 m. Roraima’s summit area is ca. 35 km^2^, which extend on three countries, Venezuela, Guyana and Brazil [55]. Our sampling sites were all located on the southern part of the summit, in Venezuela (Fig. 1C). Roraima is one of the few tepuis i.e. accessible by foot without technical climbing and is visited by thousands of international tourists every year [21, 23, 56], with an estimate of minimum 3000 in 2024 alone (Juan Mora, pers. comm.). Its pioneering vegetation grows on acidic, oligotrophic sandstone soils and the summit endures extreme and seasonally fluctuating environmental conditions [27, 55]. Sampling was carried out in two ecologically contrasting sites: one highly affected by tourism (mostly in an area locally known as “The Jacuzzis” and along the main trail leading to it from the summit entrance), representing anthropogenic disturbance (hereafter “A” samples), and the other a genuinely pristine environment representing entirely naturogenic conditions, inaccessible by established trails and showing no evidence of prior human disturbance (“N” samples) (Fig. 1C). The Jacuzzis area is one of the most prominent tourist landmarks on the summit (Fig. 2A) and is visited by the majority of those who ascend to the mountaintop. Roraima-tepui has shifted from being a sacred place, ritually protected from casual human intrusion [57, 58], to primarily serving as a sustained source of tourism-derived revenue for the region, and human waste and other detritus (Fig. 2B and C) are routinely found in the area.

### Sampling

Sterile swabs (Qiagen 4N6FLOQSwabs®) were used to swab the venter and dorsum of 20 specimens of *O. quelchii* from each site (naturogenic-N and anthropogenic-A, n = 40), applying medium pressure for ca. 30 s [59]. All specimens were collected by hand, using sterile gloves, which were changed between each individual to prevent cross-contamination. Swabs were immediately placed in 2 ml of sterile phosphate-buffered saline (PBS) in a sterile Eppendorf vial and stored at 4–8°C until processing. In addition, we sampled the soil surface (ca. 10–20 grams) next to 10 swabbed individuals from each site (n = 20). Soil samples were placed in sterile Falcon tubes and stored at 4–8°C until processing. Finally, 10 swabbed specimens from each site (n = 20) were isolated in individual sterile plastic containers and monitored until defecation for subsequent faecal sampling. Faeces were collected using sterilised surgical forceps and immediately placed in 2 ml of sterile PBS in a sterile Eppendorf vial and stored at 4–8°C until processing (see Table 1 for details about samples and geographical coordinates).

**Table 1:** Samples used in this study.

Sample (anthropogenic)	Coordinates	Sex	Skin swab	Soil	Faeces
A01	N5° 10′ 30.5” W60° 46′ 52.5”	Male	x	x	x
A02	N5° 10′ 30.7” W60° 46′ 52.5”	Male	x	x	x
A03	N5° 10′ 31.0” W60° 46′ 52.1”	Juvenile	x		
A04	N5° 10′ 31.4” W60° 46′ 51.6”	Male	x	x	x
A05	N5° 10′ 31.4” W60° 46′ 51.6”	Male	x	x	x
A06	N5° 10′ 31.7” W60° 46′ 51.2”	Male	x	x	x
A07	N5° 10′ 32.0” W60° 46′ 51.3”	Male	x	x	x
A08	N5° 10′ 32.3” W60° 46′ 51.6”	Male	x	x	x
A09	N5° 10′ 32.3” W60° 46′ 51.0”	Male	x	x	
A10	N5° 10′ 32.4” W60° 46′ 50.7”	Female	x	x	x
A11	N5° 10′ 02.0” W60° 46′ 54.5”	Male	x	x	x
A12	N5° 10′ 01.3” W60° 46′ 54.9”	Male	x		
A13	N5° 10′ 00.6” W60° 46′ 53.8”	Female	x		
A14	N5° 09′ 55.3” W60° 46′ 53.6”	Male	x		
A15	N5° 09′ 55.3” W60° 46′ 53.6”	Male	x		
A16	N5° 09′ 55.3” W60° 46′ 53.6”	Male	x		
A17	N5° 09′ 55.3” W60° 46′ 53.6”	Female	x		
A18	N5° 09′ 53.7” W60° 46′ 53.7”	Male	x		
A19	N5° 09′ 53.7” W60° 46′ 53.7”	Male	x		
A20	N5° 09′ 53.5” W60° 46′ 53.7”	Female	x		x
**Sample (naturogenic)**	**Coordinates**	**Sex**	**Skin swab**	**Soil**	**Faeces**
N01	N5° 09′ 42.5” W60° 45′ 43.3”	Female	x	x	x
N02	N5° 09′ 49.3” W60° 45′ 37.3”	Female	x	x	x
N03	N5° 09′ 56.0” W60° 45′ 34.8”	Female	x	x	
N04	N5° 09′ 56.0” W60° 45′ 34.8”	Male	x		x
N05	N5° 09′ 57.0” W60° 45′ 34.0”	Male	x	x	x
N06	N5° 09′ 57.0” W60° 45′ 34.0”	Male	x		x
N07	N5° 09′ 58.2” W60° 45′ 27.7”	Male	x	x	x
N08	N5° 09′ 54.2” W60° 45′ 33.4”	Female	x	x	x
N09	N5° 09′ 53.3” W60° 45′ 33.9”	Juvenile	x	x	
N10	N5° 09′ 58.7” W60° 45′ 29.2”	Male	x	x	
N11	N5° 09′ 58.7” W60° 45′ 29.2”	Male	x		x
N12	N5° 09′ 58.7” W60° 45′ 28.7”	Male	x	x	x
N13	N5° 09′ 58.5” W60° 45′ 28.5”	Female	x	x	x
N14	N5° 09′ 58.4” W60° 45′ 28.6”	Male	x		
N15	N5° 09′ 57.3” W60° 45′ 28.1”	Male	x		
N16	N5° 09′ 47.1” W60° 45′ 35.0”	Female	x		
N17	N5° 09′ 48.6” W60° 45′ 35.8”	Female	x		
N18	N5° 09′ 49.1” W60° 45′ 35.0”	Male	x		
N19	N5° 09′ 49.1” W60° 45′ 35.0”	Female	x		
N20	N5° 09′ 49.0” W60° 45′ 35.1”	Male	x		

### Functional microbial diversity analysis using Biolog® EcoPlates™

#### Preparation of microbial inocula for Biolog® EcoPlates™ analysis

To assess the functional diversity of microbial communities, samples of soil, faeces, and amphibian skin microbiota were processed under sterile conditions and prepared for inoculation onto Biolog® EcoPlates™, following standardised protocols optimised for environmental samples [29, 60–62].

Soil: One gram of soil was transferred into a sterile 99 cm^3^ bottle containing 0.9% (w/v) NaCl solution. The suspension was shaken at 150 rpm for 30 min at 25°C to release microbial cells into the solution. Subsequently, samples were cooled at 4°C for 30 min to stabilise metabolic activity. The resulting suspensions were then filtered through 100 μm mesh cell strainers to remove solid debris that could interfere with spectrophotometric readings [29, 42].

Skin swabs: Skin swabs immersed in sterile PBS were vortexed for 30 s to dislodge microbial cells. After removing the swabs from the Eppendorf vials, the suspensions were centrifuged at 5000 rpm for 20 min to concentrate microbial biomass. The supernatant was removed by pipetting, and 200 μL of the remaining microbial pellet was resuspended in 20 ml of sterile 0.9% NaCl solution. The suspensions were shaken at 150 rpm for 30 min at room temperature and cooled for 30 min at 4°C.

Faeces: Faecal samples suspended in sterile PBS were vortexed vigorously for 30 s to ensure thorough homogenisation. The suspensions were centrifuged at 5000 rpm for 5 min at room temperature, and the supernatant was carefully removed using a pipette. To standardise the amount of faeces used for subsequent EcoPlate analysis and to compensate for the small sample size, aliquots of 200 μL of the resulting faecal pellet were resuspended in 20 ml of sterile 0.9% NaCl solution using a modified method described by [60]. This suspension was shaken at 150 rpm for 30 min at room temperature, followed by cooling at 4°C for 30 min. The final inoculum was filtered through a 100 μm mesh cell strainer to eliminate particulate matter [29, 39].

To minimise changes in microbial activity during storage or handling, soil, faeces and skin microbial inocula were used immediately for Biolog® EcoPlates™ inoculation (as described below).

#### Biolog® EcoPlates™

The functional diversity of microorganisms in the soil, faeces and amphibian skins was determined using Biolog® EcoPlates™ (Biolog Inc., Hayward, CA, USA). The metabolic potential of substrate microbial communities was assessed using 31 different carbon sources in triplicate (each at a concentration of 1.1 g/L) categorised into six groups, including two A (putrescine and phenylethyl amine), six AA (L-arginine, L-asparagine, L-phenylalanine, L-serine, L-threonine and glycyl-L-glutamic acid), 10 CA (pyruvic acid methyl ester, D-glucosamic acid, D-galactonic acid gamma-lactone, D-galacturonic acid, 2-hydroxy benzoic acid, 4-hydroxybenzoic acid, gamma-hydroxy butyric acid, itaconic acid, alfa-ketobutyric acid and D-malic acid), four CCS (Tween 40, Tween 80, alfa-cyclodextrin and glycogen), seven CH (D-cellobiose, alfa-D-lactose, beta-methyl-Dglucoside, D-xylose, i-erythritol, D-mannitol and N-acetyl-D-glucosamine), and two PC sources (glucose-1-phosphate and D,1-alfa-glycerol phosphate). A description of the carbon sources used in the Biolog® EcoPlates™ is listed as a supplementary file.

The EcoPlates™ were inoculated with soil, faeces or skin microorganisms by pipetting 120 μL of each sample inoculum (see above) into the well plates. Samples were then incubated for 120 h at 27°C. The results were read every 24 h using the Multiskan™ Skyhigh Microplate Spectrophotometer (Thermo Scientific™). The consumption of carbon sources by the soil microbial community was determined by measuring the reduction of colourless tetrazolium chloride to red formazan (λ = 590 nm). The most significant differences in carbon substrate metabolism were observed after 72 h of incubation, and these results were used for further calculations of AWCD, H′, S, and E indices [29, 35–37, 62–66].

The overall functional diversity of microbial communities was expressed as the AWCD, calculated according to [62], as follows:


$$ \mathrm{AWCD}=\frac{\sum_{\mathrm{i}=1}^{31}\left({\mathrm{OD}}_{\mathrm{i}}-{\mathrm{OD}}_{\mathrm{blank}}\right)}{31} $$

where ${\mathrm{OD}}_{\mathrm{i}}$ is the optical density (λ = 590 nm) of each substrate well and ${\mathrm{OD}}_{\mathrm{blank}}$ is the mean optical density (λ = 590 nm) of the three control wells containing water (no carbon source). Negative values of (${\mathrm{OD}}_{\mathrm{i}}-{\mathrm{OD}}_{\mathrm{blank}}$) were set to zero prior to analysis. The AWCD serves as an indicator of the overall potential metabolic activity of the microbial community, providing an assessment of the total bioactivity for the Biolog® plates [35, 37].

The AWCD index was further divided into specific substrate groups based on their chemical nature, i.e. A, AA, CA, CCS, CH, and PC. This division of AWCD allowed for a more detailed understanding of the potential of the microbial community to degrade these carbon groups [34, 35, 67].

The H′ was applied to quantify functional diversity, reflecting the distribution and intensity of carbon substrate utilisation across the 31 substrates. It reflects the physiological diversity of bacterial communities, with higher values indicating metabolically active microbial communities capable of degrading a broader range of substrates. This approach is widely used in Biolog® EcoPlate™ analyses [62] to describe the metabolic versatility of microbial communities, analogous to its application in species-level diversity assessments. It was calculated as follows:


$$ {\mathrm{H}}^{\prime }=-\sum_{i=1}^{31} pi\ \ln (pi) $$

Where 


\begin{equation*} pi=\frac{{\mathrm{OD}}_{\mathrm{i}}-{\mathrm{OD}}_{\mathrm{blank}}}{\sum_{\mathrm{i}=1}^{31}\left({\mathrm{OD}}_{\mathrm{i}}-{\mathrm{OD}}_{\mathrm{blank}}\right)} \end{equation*}

The S index provides insight into species richness based on the number of consumed substrates, offering an additional dimension for assessing microbial diversity and activity in the soil, faeces and skin microbiomes. The S index was calculated as the number of carbon sources with positive utilisation, i.e. wells where (${\mathrm{OD}}_{\mathrm{i}}-{\mathrm{OD}}_{\mathrm{blank}}$) > 0.25.

The E index, on the other hand, relates to the relative utilisation of bacterial substrates, demonstrating the distribution pattern of microbial functional activity. A higher E index suggests that all carbon substrates are consumed at a similar rate. The E index was calculated as follows:


$$ \mathrm{E}=\frac{{\mathrm{H}}^{\prime }}{\ln \left(\mathrm{S}\right)} $$

#### Statistical analyses

To assess differences in functional microbial diversity among soils, faeces, and toads’ skin microbiomes collected from naturogenic and anthropogenic locations on the summit of Roraima-tepui, a comprehensive, multistep statistical framework was employed.

Prior to hypothesis testing, the distributions of key measured variables, including AWCD, relative utilisation of carbon substrate groups, heatmap intensities, Shannon diversity, evenness, and species richness indices, were evaluated for normality using the Shapiro–Wilk test. Homogeneity of variances across groups was assessed using Levene’s test.

Based on these preliminary diagnostics, the non-parametric Mann–Whitney U test was applied to compare central tendencies of diversity and metabolic indices between naturogenic and anthropogenic groups. This test is particularly suitable for ecological datasets, which often deviate from normality and include zero-inflated values. To complement these results and detect broader distributional changes, the Epps-Singleton two-sample test was applied. This test compares the full empirical distributions of two groups, capturing differences in location, scale, and shape, and is especially valuable in ecological studies with complex, multivariate data. Additionally, the Kolmogorov–Smirnov test was used as a supplementary method, particularly in cases of limited sample size (e.g. soil samples), offering sensitivity to variations in distribution shape and cumulative frequency. These combined tests enabled the detection of subtle yet ecologically meaningful shifts in microbial metabolic profiles that may not be apparent from mean-level comparisons alone.

To explore multivariate patterns of microbial function, a principal component analysis (PCA) was conducted on standardised absorbance data. Prior to performing the PCA, the raw data was standardised by subtracting the mean value from the data and dividing by the standard deviation. PCA biplots were generated to visualise clustering patterns by environmental origin (naturogenic vs. anthropogenic) and to identify carbon substrates and diversity indices contributing most strongly to observed group separations. Interpretation was based on the principal axes explaining the greatest variance.

This integrated and assumption-aware statistical strategy enabled a robust and nuanced evaluation of environmental impacts on functional diversity of soil, faeces and toads’ skin microbiomes. All statistical analyses were performed using the software PAST 4.3 [68] at a significance threshold of 0.05.

## Results

### Comparative assessment of overall and group-specific average well colour development

**Figure 3:**
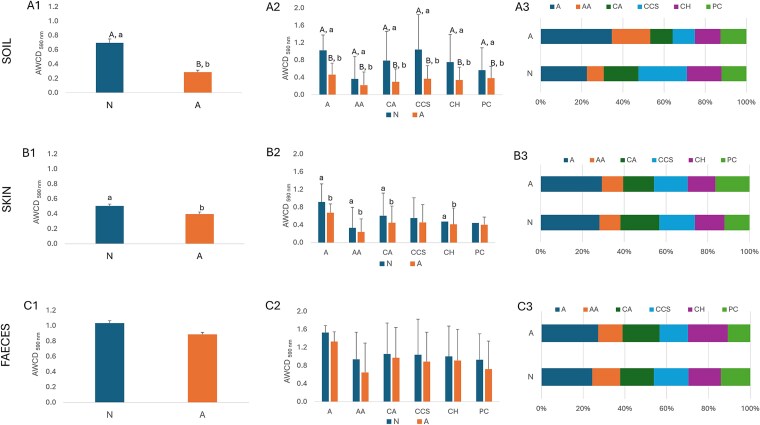
Functional microbial diversity of soil (A1–A3), skin (B1–B3), and faecal (C1–C3) microbiomes in samples collected from naturogenic (N) and anthropogenic (A) areas, based on Biolog® EcoPlates™ analysis. Panels A1, B1, and C1 show average well colour development (AWCD) as an indicator of overall metabolic activity. Panels A2, B2, and C2 present substrate utilisation profiles across different carbon source groups. Panels A3, B3, and C3 display the relative (%) contribution of each carbon group to the total AWCD. Substrate groups: A- amines, AA- amino acids, CA- carboxylic acids, CCS- complex carbon sources, CH- carbohydrates, PC- phosphate carbon. Statistical differences in median values between sample types (N vs A) based on the Mann–Whitney U test are indicated by different capital letters (A, B) (*P* ≤ .05); differences in distribution patterns are marked by lowercase letters (a, b), as determined using the Kolmogorov–Smirnov and Epps-Singleton tests (*P* ≤ .05).

A comparative assessment of microbial community functional diversity in soil, skin, and faecal samples collected from naturogenic (N) and anthropogenically impacted (A) areas is presented in Fig. 3.

In soil samples (Fig. 3A1), overall AWCD was significantly lower (decrease of 59%) in samples from the anthropogenically impacted area, both in terms of median values and distribution patterns, as confirmed by Mann–Whitney U and Kolmogorov–Smirnov tests (*P* ≤ .05), indicating reduced overall functional metabolic potential (Fig. 3A1). Functional group analysis (Fig. 3A2) revealed that this reduction affected all carbon source groups, with decreases of 32% for phosphate carbon (PC), 39% for amino acids (AA), 55% for amines (A) and carbohydrates (CH), 62% for carboxylic acids (CA), and 65% for complex carbon sources (CCS). The relative contribution plot (Fig. 3A3) showed a shift in metabolic profiles in A samples, with increased utilisation of A and AA, suggesting an altered microbial structure or selection for stress-tolerant taxa. In contrast, N samples showed higher utilisation of CCS, typically associated with more functionally diverse and stable microbial communities in undisturbed environments.

Skin microbiomes (Fig. 3B1) also showed reduced overall AWCD in A samples (decrease of 21%), although the differences were moderate and less pronounced than in soils. The statistical analysis indicated significant differences in distribution between N and A (Kolmogorov–Smirnov and Epps-Singleton tests, *P* ≤ .05). Functional group patterns (Fig. 3B2) were more consistent than in soils but still revealed significant reductions in A (26%), AA (27%), CA (26%), and CH (13%), with differences in distribution confirmed by the Kolmogorov–Smirnov and Epps-Singleton tests (*P* ≤ .05). Unlike soil samples, the relative contributions of amines and amino acids in N and A samples remained stable (Fig. 3B3), indicating a higher degree of functional resilience in skin-associated microbiomes under anthropogenic influence.

Faecal microbiomes (Fig. 3C1) exhibited the highest overall AWCD values among all sample types, with only minimal and statistically nonsignificant differences between N and A (decrease of 14%). Functional group-specific utilisation (Fig. 3C2) and relative contribution profiles (Fig. 3C3) showed slightly lower values in A samples; however, these differences were not statistically significant. These findings suggest that the faecal microbiome demonstrates a high degree of functional stability and resilience, maintaining a consistent metabolic profile irrespective of environmental conditions.

The abovementioned results highlight that microbial communities in soil are the most sensitive to anthropogenic impact, showing both reduced functional microbial diversity and altered substrate utilisation. Skin microbiomes show intermediate sensitivity, while faecal microbiomes appear resilient, maintaining consistent metabolic profiles across the two different environmental contexts.

### Substrate utilisation patterns

**Figure 4:**
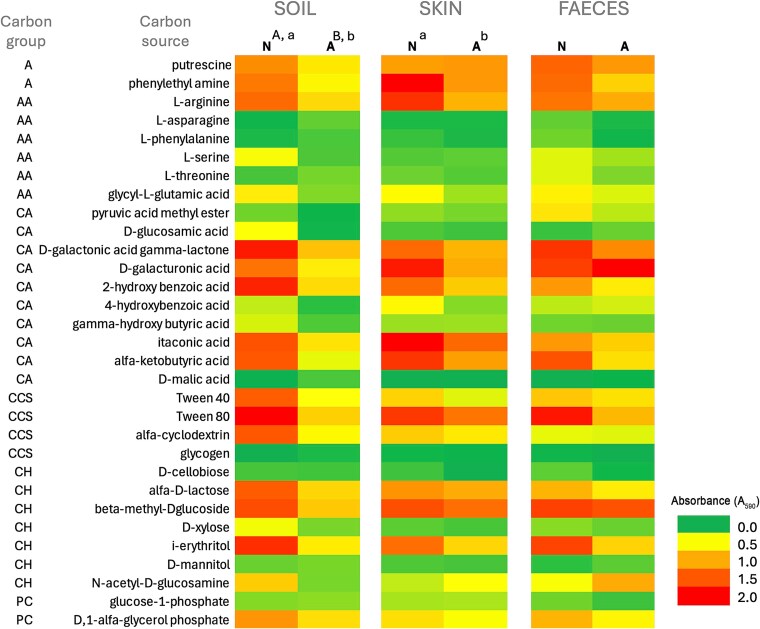
Heatmaps presenting the intensity of carbon source utilisation in soil, skin, and faecal microbiomes collected from naturogenic (N) and anthropogenic (J) areas. Substrate groups: A- amines, AA- amino acids, CA- carboxylic acids, CCS- complex carbon sources, CH- carbohydrates, PC- phosphate carbon. Colours indicate the relative utilisation intensity of individual carbon sources, reflecting metabolic activity patterns across the N and A samples. Statistical differences in median values between sample types (N vs A) based on the Mann–Whitney U test are indicated by different capital letters (A, B) (*P* ≤ .05); differences in distribution patterns are marked by lowercase letters (a, b), as determined using the Kolmogorov–Smirnov and Epps-singleton tests (*P* ≤ .05).

In addition to the above results, Fig. 4 further illustrates the differences in microbial substrate utilisation patterns across soil, skin, and faecal microbiomes from N and A areas. These heatmaps display the relative intensity of carbon source utilisation across 31 substrates. More intense colours (red) reflect higher utilisation of specific carbon substrate.

Consistent with the earlier findings, soil microbiomes from N areas demonstrated notably higher substrates utilisation, particularly within the CCS, CH, and CA groups. These samples exhibited a broader and more intense metabolic profile compared to those from human-impacted areas. The differences in both median values and distribution patterns between N and A soils, confirmed by the Mann–Whitney U and Kolmogorov–Smirnov tests (*P* ≤ .05), highlight a substantial decline in microbial functional diversity and activity under anthropogenic pressure.

Skin microbiomes showed more moderate shifts in carbon sources utilisation. While certain substrates within the A, AA, and CA groups exhibited reduced utilisation in A samples, others, such as those from CH and PC groups, remained relatively stable. The observed differences in distribution patterns between N and A samples (as indicated by statistical markers) suggest that skin-associated microbiomes are affected by environmental disturbance, though to a lesser extent than soil communities.

In contrast, faecal microbiomes displayed high and relatively uniform utilisation across both environments. No statistically significant differences were observed in either median values or distribution patterns, indicating strong functional resilience. These communities appear largely unaffected by external environmental variation, maintaining stable metabolic profiles regardless of sampling location. Overall, the heatmap analysis (Fig. 4) reinforces previous observations: soil microbiomes are the most vulnerable to anthropogenic impacts, followed by skin microbiomes, while faecal microbiomes remain functionally stable across environmental gradients.

### Diversity indices

**Figure 5:**
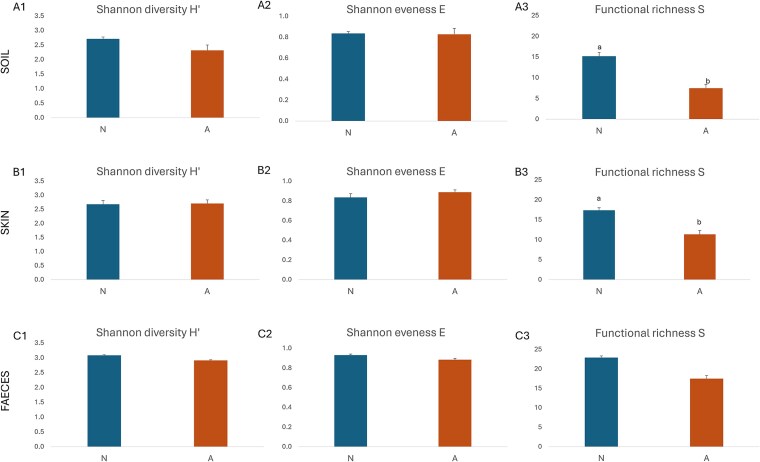
Microbial catabolic diversity indexes calculated for the data obtained from the Biolog® EcoPlates™. Different letters (a, b) indicate significant differences in distribution patterns according to Kolmogorov–Smirnov and Epps-singleton tests (*P* ≤ .05).

The patterns reported above are mirrored in the diversity metrics (Fig. 5). In soil microbiomes (Fig. 5A1–A3), a noticeable decline in H′ and especially in S index is observed in A samples compared to N, confirming that anthropogenic disturbance substantially reduces both the number and functional variety of microbial taxa. Interestingly, E index is slightly higher in A samples, likely reflecting a more uniform but reduced microbial community structure, often observed when sensitive taxa are lost and dominance shifts to a few stress-tolerant species.

Skin microbiomes (Fig. 5B1–B3) also exhibit a reduction in S index and a slight drop in H′ in A samples, consistent with previous observations of moderate functional loss in impacted areas. As with soil, evenness increases under anthropogenic influence, suggesting that disturbance may simplify the microbial structure, favouring more evenly distributed but functionally narrower communities.

In faecal microbiomes (Fig. 5C1–C3), the diversity metrics show the smallest differences between N and A samples. H′ and E indices are nearly identical, while S index shows only a modest, and statistically irrelevant decrease in impacted area. These results align with earlier findings indicating high functional resilience and stability of faecal microbiomes.

### Principal component analysis

**Figure 6:**
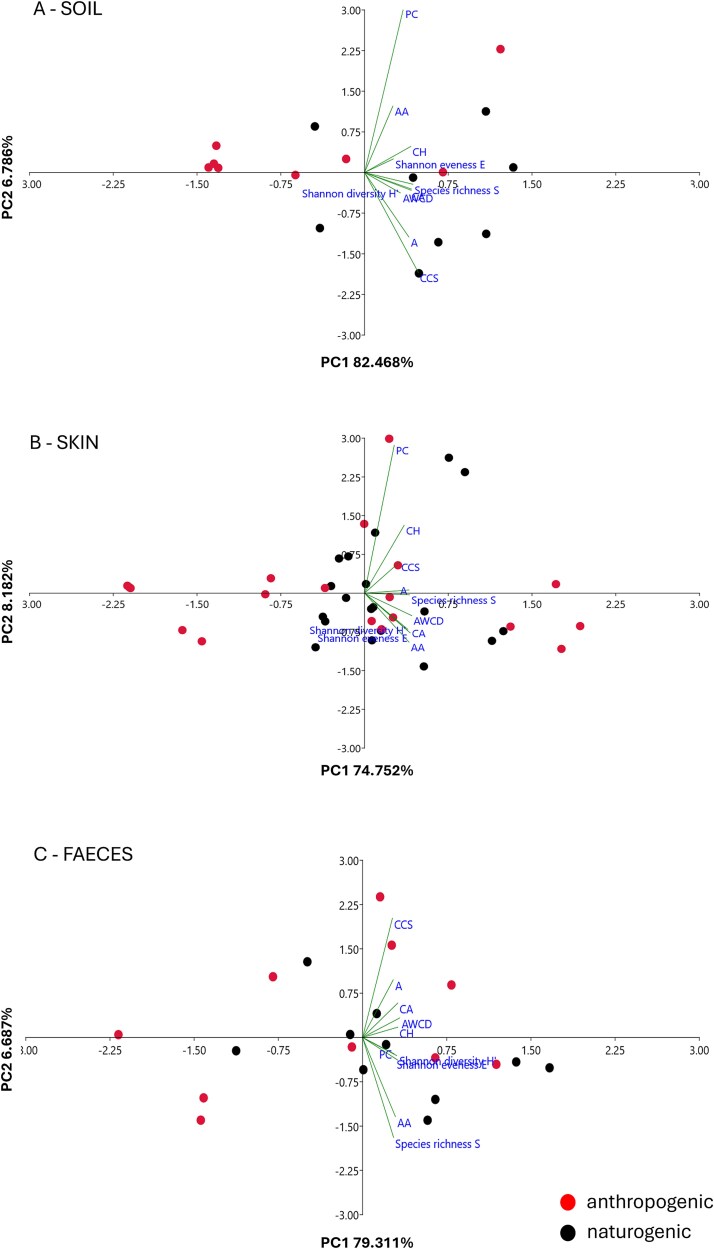
Principal component analysis (PCA) with functional microbial diversity parameters of soil (A), skin (B) and faeces samples (C) collected from naturogenic (black dots) and anthropogenic (red dots) locations. AWCD – average well colour development, A- amines, AA- amino acids, CA- carboxylic acids, CCS- complex carbon sources, CH- carbohydrates, PC- phosphate carbon.

PCA further visualises the above trends (Fig. 6). For the soil samples (Fig. 6A), the first two principal components (PC1 and PC2) explained 89% of the variation in substrate utilisation profiles across the two sample groups. PC1 accounts for the majority of this variation (82.47%) and effectively distinguishes soil samples from N versus A sites. This indicates that anthropogenic disturbance is the primary factor driving functional differences in the soil microbial communities. PC2 explains a smaller proportion of variance (6.79%) and likely reflects more subtle within-group variability or specific changes in the utilisation of certain substrate categories. The clustering pattern observed in the PCA aligns with earlier results from AWCD and heatmap analyses, confirming that microbial communities in impacted soils exhibit markedly reduced functional diversity and substrate utilisation compared to natural soils. Examination of the loading plot reveals that all carbon substrates have positive loadings on PC1, suggesting that this component primarily represents overall substrate utilisation intensity. Notably, substrates from the CA and CCS groups exhibit the highest positive loadings and are closely associated with the N samples positioned on the positive side of PC1. This pattern indicates that the enhanced functional diversity and broader substrate use in N samples drive their separation from A soils along PC1. In contrast, the A samples cluster on the negative side of PC1, reflecting a general decline in substrate utilisation intensity rather than a shift toward alternative substrates.

In skin microbiomes (Fig. 6B), the PC1 and PC2 explain 83% of the total variance, with PC1 accounting for the majority at 74.75%. A partial separation between N and A samples is evident, although less distinct than in soil communities. Samples from the N sites cluster more tightly together, indicating greater functional similarity, whereas samples from A sites are more dispersed throughout the plot, reflecting higher variability in substrate utilisation. The loadings show correlations with substrates utilised by both sample types, reflecting an intermediate clustering pattern. This finding aligns with earlier analyses that identified moderate but statistically significant reductions in the utilisation of specific substrate groups in impacted skin microbiomes.

By contrast, faecal microbiomes (Fig. 6C) from N and A environments form a single, overlapping cluster, demonstrating functional stability and consistency across environmental contexts. This strongly supports the earlier finding that faecal microbiomes are minimally influenced by external anthropogenic pressures and maintain a conserved functional profile regardless of collection site.

Altogether, this multivariate analysis visually confirms the gradient of anthropogenic sensitivity observed across microbiomes: soil > skin > faeces, supporting the conclusion that anthropogenic disturbance on the tepui summit shapes microbial functional diversity in this fragile toad-habitat ecosystem.

## Discussion

Human-driven environmental change is accelerating the global loss of biodiversity [1–4], yet its effects on microbial life, despite microbes’ fundamental role in sustaining ecosystems [6], remain insufficiently explored. While the decline of visible species and habitats is well documented, disruptions to microbial communities and their ecological functions are less apparent but potentially far-reaching. Because microorganisms mediate key biogeochemical processes and support ecosystem resilience [6], understanding how anthropogenic pressures alter microbial diversity and function is critical for predicting broader ecological consequences.

In the present study, soil samples from anthropogenically impacted areas exhibited lower AWCD (overall and related to particular carbon groups) and reduced diversity indices, indicating a decline in overall microbial functional diversity, which is consistent with a plethora of studies demonstrating the negative impact of anthropogenic changes, such as urbanisation or pollution, on microbial metrics derived from Biolog® EcoPlates™ [69–72]. Significantly, microbial communities in anthropogenically impacted environments often exhibit lower substrate richness, i.e. the number of different carbon sources utilised, suggesting a reduced functional diversity and narrower metabolic potential. This pattern was consistently observed in polluted environments, where microbial communities face ecological stress due to chemical or organic pollutants. For example, soils exposed to heavy metal contamination demonstrated significantly lower functional richness and reduced AWCD compared to less polluted controls [11]. Similarly, microbial communities from highly polluted forest soils showed diminished EcoPlate-derived diversity indices, including both substrate richness and AWCD, relative to reference sites [70].

These trends extend to aquatic systems as well. In an assessment of river and lake ecosystems, sediments from less-polluted upstream river sites utilised nearly all 31 carbon substrates (richness ca. 29–31), whereas polluted downstream lake sediments exhibited markedly reduced substrate utilisation, lower AWCD, and fewer utilised substrates [71]. Further evidence from ecosystems affected by acid mine drainage, tannery discharge, and refinery effluents confirms that pollution reduces both substrate richness and metabolic rates, indicating a loss of functional complexity [72].

Despite this general reduction in AWCD and substrate richness, a notable increase in the utilisation of amines and amino acids was observed in samples from the human-impacted area compared to the natural reference site (Figs 3–4). These two carbon source groups serve as important organic nitrogen substrates for microbial communities. Their elevated utilisation suggests enhanced microbial involvement in nitrogen cycling processes, such as ammonification and nitrogen assimilation. This indicates a shift in microbial functional activity toward nitrogen transformation pathways, likely reflecting the increased availability of nitrogen-rich compounds in polluted or nutrient-enriched environments [70, 72, 73]. Such a response is consistent with conditions commonly found in human-impacted ecosystems, where organic waste inputs elevate nitrogen substrate availability and select for metabolically specialised or copiotrophic microbial taxa.

Notably, increased utilisation of amino acids and amines is frequently associated with copiotrophic microbial taxa, which thrive in nutrient-rich environments [73]. Such functional profiles are often observed in ecosystems influenced by anthropogenic inputs, such as urban runoff, wastewater-impacted soils, or areas contaminated by faecal and urine-derived waste. Indeed, microbial communities in soils amended with municipal sewage sludge show significantly higher utilisation of amino acids and amines compared to unpolluted soils, reflecting elevated organic nitrogen levels [70]. These findings confirm that elevated amino acid metabolism in EcoPlate assays can act as a reliable biomarker of human-derived organic contamination. Collectively, these findings underscore the vulnerability of microbial metabolic diversity to anthropogenic pressures. While some microbial taxa may flourish in nutrient-enriched or contaminated conditions, especially those capable of utilising nitrogen-rich compounds, the overall loss of substrate richness, diminished carbon group utilisation, and reduced AWCD suggest a shift toward functional simplification and ecological stress. Thus, the EcoPlate approach proves effective not only for functional profiling but also as a sensitive indicator of environmental degradation and pollution(human)-driven shifts in microbial communities.

The reduced AWCD and lower substrate richness was also evident in the skin microbiome of *O. quelchii* collected from human-impacted areas. This pattern mirrors the findings observed in soil samples and suggests that human-induced environmental stressors not only alter the surrounding microbial habitat but also extend their influence on the host-associated microbiota of resident organisms. The diminished metabolic diversity on amphibian skin may reflect reduced microbial resilience and functional redundancy, potentially compromising host health and ecological fitness. These results emphasise the broader ecological consequences of anthropogenic disturbance (in our particular case, tourism in a small, insular fragile ecosystem), highlighting that human activities affect both environmental and symbiotic microbial communities. Further research is needed to elucidate the link between the observed shift in microbial functional profiles in the skin microbiome of *O. quelchii* at summit sites most impacted by tourism (see Fig. 1C) and the recent detection of *Bd* in the same environmental context [21].

Our finding that *O. quelchii* faecal microbiomes are functionally stable and consistent across anthropogenic vs naturogenic contexts align with those of, e.g. [74], who demonstrated that, in rodents, gut microbiota composition is shaped by both current and historical habitats, with certain characteristics of the microbiome reflecting earlier environmental conditions. This suggests a relative stability of faecal microbiomes, which can persist despite environmental disturbances. However, it is important to note that gut and faecal microbial communities predominantly inhabit anoxic or microaerophilic environments, where many strict anaerobes simply cannot grow (and may rapidly die) upon exposure to oxygen. Accordingly, the metabolic pathways expressed *in vivo* differ markedly from those detectable under aerobic laboratory conditions [50, 75, 76]. Our reliance on an aerobic incubation protocol with the Biolog® EcoPlate™ therefore constitutes an important methodological limitation as the metabolic profiles obtained largely reflect the oxygen-tolerant subset of the faecal microbiota and fail to capture the full functional potential of obligate anaerobes [50, 76]. Nevertheless, this limitation does not compromise the validity of our comparisons because anthropogenic and naturogenic populations were assessed using the same protocol ensuring that relative differences among faecal microbiota are preserved even if only a subset of taxa remained metabolically active. Notably, Biolog® also provides dedicated anaerobic profiling systems for characterising these taxa.

Our results also point to the importance of including microbial measures in biodiversity monitoring frameworks, particularly in sensitive or poorly studied ecosystems, highlighting the utility of the EcoPlates™ in detecting changes in microbial communities associated with amphibians and their microhabitats.

## Conclusion

Our study provides evidence of shifts in microbial functional profiles in soil and host-associated microbiomes (particularly the skin of *O. quelchii*) under anthropogenic disturbance relative to pristine sites. These findings raise significant concern notably because such alterations of the microbial composition could disrupt host immunity and disease resistance in a unique, ecologically delicate ecosystem where *Bd* has recently been detected [21]. As already emphasised [21, 23], we call for immediate measures to mitigate anthropogenic disturbance on the summit of Roraima-tepui. While Roraima contributes steadily to regional tourism income [56], poor long-term planning and limited concern for the cumulative impacts of anthropogenic pressure threaten its ecological integrity and may ultimately lead to the extirpation of its unique biodiversity. We recommend limiting tourist access to the summit, mandating the use of portable sanitation facilities for all individuals, including guides and porters (currently only mandatory for tourists), enforcing strict penalties for littering (including for guides and porters), and designating extensive areas of the summit as off-limits to human activities to minimise ecological disturbance.

## Data Availability

Data supporting the analyses in this contribution are available on figshare at https://doi.org/10.6084/m9.figshare.29775917.
